# Online Sex Addiction: A Qualitative Analysis of Symptoms in Treatment-Seeking Men

**DOI:** 10.3389/fpsyt.2022.907549

**Published:** 2022-07-07

**Authors:** Lukas Blinka, Anna Ševčíková, Michael Dreier, Katerina Škařupová, Klaus Wölfling

**Affiliations:** ^1^Institute for Research on Children, Youth, and Family, Faculty of Social Studies, Masaryk University, Brno, Czechia; ^2^Outpatient Clinic for Behavioral Addictions, Department of Psychosomatic Medicine and Psychotherapy, University Medical Center of the Johannes Gutenberg-University Mainz, Mainz, Germany

**Keywords:** pornography addiction, hypersexuality, Compulsive Sexual Behavior (CSB), addiction symptoms, qualitative study, treatment-seeking sample

## Abstract

**Background:**

Problematic sexual internet use has been attracting increasing research attention in recent years. However, there is a paucity of qualitative studies about how this problem manifests on a daily basis in the clinical population and whether the phenomenon should fall within the hypersexual, compulsive-impulsive, or addictive spectrums of disorders.

**Methods:**

Twenty-three semi-structured interviews, including AICA-C clinical interviews, were conducted with men who were in treatment for problematic internet sex use (aged 22–53; Mage = 35.82). The interview structure focused on the patterns of sexual behavior in question, their development, the manifestation of symptoms, and other associated psychosocial problems. A thematic analysis was applied as the main analytical strategy.

**Results:**

Typical problematic patterns included pornography use and cybersex, together with continuous masturbation for several hours several times a week. This pattern emerged relatively early in young adulthood and became persistent for years. The majority of participants fulfilled the criteria for behavioral addiction (as defined, e.g., by the components model of addiction), with loss of control and preoccupation being the most pronounced and withdrawal symptoms being the least. Together with the onset of erectile dysfunction, negative consequences were reported as being slowly built up over years and typically in the form of deep life dissatisfaction, regret, and feelings of unfulfilled potential.

**Discussion and Conclusion:**

The Addiction model is relevant for describing the difficulties in treatment-seeking men who suffer from problematic sexual internet use. However, the manifestations of the additional criteria are nuanced. In the case of negative consequences, their onset might be very slow and not easily reflected. While there was evidence of several forms of tolerance, potential withdrawal symptoms in online sex addiction need further attention to be verified.

## Introduction

Internet use for sexual purposes has rendered various opportunities and effects. Internet users may benefit from searching for sexual information, searching for sexual partners, or exploring and fulfilling sexual needs (1, 2). Nonetheless, several risks emerged (3). One of the most discussed risks has been labeled excessive, out-of-control, problematic, compulsive, or addictive use of the internet for sexual purposes (4, 5). These terms are often understood to be synonymous. In this text, we use Problematic Sexual Internet Use [PSIU, (6)] as an umbrella term. Despite a rapidly growing number of recently published studies, the available literature has several limitations. Our knowledge is mostly derived from the findings of survey-type research conducted in the general population. We have rather limited information from other types of studies, including qualitative studies, that would examine such problematic internet use in a clinical or sub-clinical sample and, specifically, for people who have sought help for their behavior (5). Increasing evidence for this subpopulation is highly needed due to controversies that guide the conceptualization of the phenomenon, specifically whether and to what extent the addiction model is applicable in this problematic behavior, or whether it is better to use the classification of the compulsive-impulsive spectrum of disorders (7), which is a distinction that has important implications for the treatment approach. Moreover, the analysis of the symptoms reported by those in treatment for PSIU is essential for the better understanding of the condition, the improvement of diagnostic guidelines, and better-targeted treatment approaches.

In the existing literature, there are several theoretical conceptualizations related to PSIU. These include hypersexual, addiction, and compulsive models. All are umbrella terms for various non-paraphilic problematic behaviors, which range from online and offline pornography use, cybersex, and telephone sex, and which result in excessive masturbation or other forms of sexual behavior with consenting adults. The concept of hypersexual disorder (8) received extensive attention and was proposed for inclusion in DSM-5, albeit unsuccessfully (9, 10). Later on, the International Classification of Diseases underwent revision, resulting in acknowledgment within the Compulsive Sexual Behavior Disorder [CSBD; 11] as an official disorder that belongs under the umbrella of impulse control disorders (11). The behavioral addiction model is a long-term popular conceptualization (12, 13) that has been applied to excessive sexual behavior and has not been officially acknowledged. However, the diagnosis of “Other specified disorders due to addictive behaviors” in ICD-11 (14) may be convenient, particularly in the case of problematic pornography use (15). These three models have several substantial overlaps and differences (7). First, they all describe problematic sexual behavior that is repetitive and long-term, it is a condition that cannot be explained by another major condition or another disorder, and it concerns a non-paraphilic behavior. Second, they agree on the manifestation of three main symptoms: (1) *salience* (i.e., the activity becomes dominant in one's life and interferes with fulfilling important goals and obligations, overpowering one's thinking and feeling in the form of cognitive preoccupation and craving); (2) *loss of control* or *relapse* (i.e., repeatedly unsuccessful efforts to control or reduce sexual fantasies, urges, and behaviors while disregarding the risk of physical and emotional harm to oneself or to others, and restoring the previous behavioral pattern even after a long period of abstinence); and (3) *negative consequences, conflicts*, or *problems* (i.e., the activity brings personal distress or impairment in social, occupational, or other important areas of life). In terms of differences, only the addiction and hypersexual models point to the (4) *mood regulation* component, which is pleasure-seeking behavior that attempts to elevate one's mood. The compulsive model considers the problematic behavior rather connected to decreasing anxiety and does not see it as reward-pleasure seeking, *per se*. Further, only the addiction model includes two additional criteria: (5) *tolerance* (i.e., the over-time tendency to experience less or none of the pleasurable effects that stem from the activity), and (6) *withdrawal symptoms* (i.e., unpleasant feelings when the behavior cannot be performed).

Some of the symptoms and manifestations of PSIU are relatively well described in the literature. These include negative consequences (16–19), loss of control (20), mood management (21, 22), and salience/preoccupation (23). However, relatively weaker evidence exists for tolerance and withdrawal symptoms, and their manifestations. For instance, Schneider (24) described how cybersex may escalate and quickly become a dominant activity. Wines (17) showed that some members of Sex Addicts Anonymous tend to increase their problematic behavior after relapse. Some evidence shows that depression, anger, anxiety, insomnia, fatigue, increased heart rate, disorientation, confusion, numbness, and the inability to focus or concentrate–all states described by patients–could be signs of withdrawal symptoms (17, 25). However, these experiences were reported in relation to offline, rather than online (PSIU), sexual behavior. Moreover, Sassover and Weinstein (26) critically point to the lack of empirical evidence as to whether these feelings could be explained by withdrawal or, rather, if they represent preceding dysphoric states.

Furthermore, some scholars (27) doubt the very existence of withdrawal symptoms and tolerance in behavioral addictions, in general. Criticism has been specifically raised against the application of the addiction model to the out-of-control use of the internet for sexual purposes, which has been deemed inappropriate due to a lack of evidence for the presence of all six addiction-model components (26, 28, 29). Moreover, a large number of studies [see (30, 31) for systematic review] found that religiosity or moral incongruence may affect the perceptions of one's own behavior and ultimately lead to the overestimation of the problem and an inappropriate (self) diagnosis. On the other hand, Gola et al. (32) stated that moral incongruence (i.e., the potential religiosity behind it) is culturally influenced and its status as exclusion criteria for PSIU is questionable. A lack of qualitative studies on the manifestations of PSIU in different cultural contexts may result in a misunderstanding of the problems due to which some people seek help.

Despite the rapidly growing body of research in recent years (5), there are still uncertainties about the conceptualization of PSIU (26, 33). As suggested (34), when there is an uncertain status for any potential behavioral addiction, a person-centered (i.e., qualitative) approach is needed to explore its phenomenology and etiology. Thus, the present study aims to describe the lived experience of men in treatment for their PSIU. The main goal is to analyze and describe the perceived manifestation of symptoms, their development over time, and the associated psychological and health issues. This, subsequently, enables us to confront the results with the existing classifications in the literature–whether they can be addressed as part of the addiction model or with the hypersexual or compulsive models.

## Materials and Methods

### Sample and Participants

Adult men (aged ≥ 18) who had an experience with treatment PSIU were included in the study. Because there are no specialized sex addiction or behavioral addiction treatment centers in the Czech Republic, we searched online for active professionals (i.e., clinical psychologists, psychotherapists, practitioners) with the focus on sexology and addictology. In total, 104 practitioners were contacted and asked whether they had such patients and whether they would invite them to participate in the research. Due to the lower effectiveness of this style of recruitment, we also contacted Czech and Slovak self-help groups (because the two countries are interconnected due to the shared history and mutually comprehensive languages). Specifically, the Sex Addicts Anonymous (SAA) and Sexaholics Anonymous (SA) networks were proffered a request to participate in the research. Upon further analysis, we included only those SAA and SA members who were also undergoing professional treatment.

The sample characteristics are shown in Table 1. The overall study sample included 23 men 22–53 years old (Mage = 35.82 years, SD = 7.54, Median = 34; 6 of which were of Slovak nationality, 26%). The sample can be characterized as rather well-educated, with only one man having only elementary education and 15 participants (65%) having achieved a college or university level of education. Sixteen participants were married or engaged at the time of the interview, six were divorced, and one was widowed. Seven participants were religious (Roman Catholic), and four of them confirmed that the use of pornography conflicted with their religion (P5, P7, P9, P14; *n* = 4; 17%). All of the participants, except one, self-identified as heterosexuals. We also asked about having a history of other forms of addiction, drug use, or mental health conditions from which the participants suffered or for which they received treatment. Only a minority of the participants (*n* = 5; 22%) reported no comorbidity with other disorders or had no further clinical or subclinical issues. The most common past or present addiction-like comorbidity included seven cases of excessive computer gaming, six cases of excessive alcohol use, four cases of amphetamine or methamphetamine use, three cases of gambling behavior, and one case of bulimia nervosa. Only a few participants reported other mental disorders, including major depression, bipolar disorder, schizoid personality disorder, and narcissistic personality disorder. It must also be noted that there was another level of comorbidity that could not be labeled as clinical, though, in the words of the respondents, it was very important for their condition–specifically, it was their perceived unattractiveness, shyness, and/or inability to confidently communicate with women (P1, P3, P5, P10, P11, P14, P15, P20, P21; *n* = 9; 39%). Upon the observation of the interviewer (a trained psychologist), a portion of the participants suggested alexithymia, another issue which manifested as the lowered ability to be aware or reflect upon one's own emotional states and to communicate them. This was very significant for Participants 4, 10, and 20 (*n* = 3; 13%), but probably played some role for other participants, too.

**Table 1:** Key characteristics of the participants.

**Subject**	**Age**	**Nationality**	**Education**	**Family status**	**Religiosity**	**Comorbidity**
1	22	CZ	HS	Engaged	N	Low self-confidence/shyness
2	34	CZ	HS	Married	N	Recreational polydrug use
3	50	CZ	U	Single	N	Bulimia nervosa, obesity; low self-confidence/shyness
4	27	CZ	C	Married	N	Alexithymia
5	36	CZ	HS	Single	Y	Excessive gaming; ecstasy and methamphetamine use; low self-confidence/shyness
6	34	CZ	U	Married	N	None
7	34	CZ	U	Married	Y	None
8	40	CZ	E	Married; children	N	Excessive alcohol use
9	29	CZ	C	Single	Y	None
10	32	CZ	HS	Single	N	Excessive alcohol use, gambling, alexithymia, low self-confidence/shyness
11	42	CZ	U	Married; children	N	Excessive gaming, low self-confidence/shyness
12	53	CZ	HS	Widowed; children	N	Excessive gaming; binge drinking; depression
13	44	CZ	U	Divorced; children	N	Excessive alcohol use
14	26	CZ	U	engaged	Y	Low self-confidence/shyness
15	40	CZ	C	Married, children	Y	Bipolar and schizoid disorder; methamphetamine use; gambling; low self-confidence/shyness
16	29	SK	U	Married	Y	Excessive gaming
17	32	CZ	U	Married; children	N	Excessive gaming; methamphetamine use
18	41	SK	U	Divorced	N	Excessive alcohol use
19	30	CZ	U	Married	N	None
20	37	SK	U	Married; children	Y	Alexithymia; low self-confidence/shyness
21	42	CZ	U	Married; children	N	Excessive gaming; low self-confidence/shyness
22	32	CZ	HS	Engaged	N	None
23	38	CZ	HS	Engaged	N	Narcissistic personality disorder; excessive alcohol use; excessive gaming; methamphetamine use

*CZ, Czech nationality; SK, Slovak nationality; E, elementary school; HS, high school; C, college; U, university; N, non-religious; Y, religious*.

### Procedure

After receiving informed consent, 13 interviews were carried out face-to-face and eight interviews were conducted online as video calls. Two participants were interviewed with an online chat. The anonymity of the participants was strictly safeguarded and all of the identification details were deleted from the interview transcriptions. The audio interviews lasted from 37 min to 2 h and 13 min. The two typed interviews via Skype chat lasted about 5 h.

A semi-structured interview protocol was created to map the three main parts. The first part included questions about the background characteristics of the participants, such as education, family situation, sexual development, other addictive behaviors, and other physical or mental issues (e.g., “*Have you ever taken drugs?*” “*If yes, when/which drugs/how often*?”). The second part included queries about the patterns of their problematic sexual behavior (e.g., questions on how it manifests, how the typical episode looked, its frequency and length, preferred content, triggering factors), the start of the problems and their development over time (e.g., “*How did you know you already had a problem?*”), the escalation of the problem, and their experience with the treatment (e.g., what facilitated the need for the treatment, how the behavior changed under influence of the treatment). The interview structure was developed to be flexible so that each problematic sexual behavior could be explored intensively. The third part (although often mixed with the second part) included the manifestation of the symptoms of the sexual behavior in question. We asked participants to describe their problematic sexual behavior and how it manifested (e.g., “*In what way has watching pornography become uncontrollable for you?*”). For this purpose, we utilized the AICA-C: a Standardized Clinical Interview to Assess Internet addiction (35). Its results are shown in Table 2. Since AICA-C is based on the criteria of behavioral addiction (i.e., craving, tolerance, withdrawal symptoms, the loss of control, preoccupation, negative consequences) but excludes mood management, the interview structure was enriched with passages that mapped mood management symptoms.

**Table 2:** Participants' characteristics in relation to problematic sexual internet use.

**Subject**	**Age of problem recognition**	**Form of problematic behavior**	**AICA-C addiction symptoms**					
			**Craving**	**Tolerance**	**Withdrawal**	**Salience**	**Consequences**	**Loss of control**
1	18	Porn, cybersex, dating websites	4	4	2	5	4	5
2	23	dating websites; Masturbation with porn	2	0	2	2	2	1
3	30	Masturbation with; dating websites; sex workers	3	4	1	4	3	5
4	24	Masturbation with porn	4	2	3	4	2	5
5	30	Masturbation with porn	2	2	1	2	2	4
6	32	Masturbation with porn	2	0	0	1	4	1
7	29	Masturbation with porn	4	5	4	4	3	5
8	32	Masturbation with porn; sex workers; cybersex	3	4	uncertain	4	4	4
9	19	Masturbation with porn	3	3	3	4	3	4
10	25	Masturbation with porn; cybersex; sex workers	4	4	4	5	3	4
11	25	Masturbation with porn	1	0	0	1	1	3
12	37	Masturbation with porn	4	5	3	4	5	5
13	25	Masturbation with porn; sex workers	4	5	3	4	5	5
14	19	Masturbation with porn	2	2	2	2	1	3
15	28	Masturbation with porn	5	3	2	3	2	2
16	16	Masturbation with porn	4	1	2	5	3	5
17	25	Masturbation with porn; serial infidelity	5	4	uncertain	4	5	4
18	Uncertain	Masturbation with porn; coercive sexual practices; sex workers	4	4	3	4	4	4
19	24	Masturbation with porn	3	2	4	3	2	4
20	30	Masturbation with porn	4	3	2	4	3	4
21	30	Masturbation with porn	3	1	1	5	2	5
22	Uncertain	Masturbation with and without porn, cybersex	4	3	4	2	2	4
23	Uncertain	Masturbation with porn	4	1	2	5	2	5

*Questions about the AICA-C symptoms were asked about the peak period. Each criterion was evaluated on a scale ranging from 0 = never/no sign to 5 = very frequent/very significant*.

### Data Analysis

In this study, we used a thematic analysis because it provides a flexible and useful research tool for identifying, analyzing, and reporting patterns (i.e., themes) within the data (36). Since the topic of the out-of-control use of the internet for sexual purposes has been intensively studied in recent years (5) through several theoretical models, we focused on the further validation and precision of these conceptualizations rather than on the creation of a new theory. Therefore, the use of a theoretical or deductive– “top-down” –approach to the data analysis is relevant and justifiable (37). The themes and patterns were derived based on the consideration of the selected theoretical framework, which includes the conceptualization of behavioral addiction because it also covers the criteria for hypersexuality and CSBD.

Prior to coding the interviews, the categories that covered the criteria of behavioral addiction in general [e.g., (38, 39)] and sex addiction in particular [e.g., (40)] were established in an Excel spreadsheet. The coding was done by the first author and supervised by the second author. We specifically focused on marking experiences that would correspond to craving, a growing tolerance to consumed sexual materials, and to withdrawal symptoms (e.g., *I started deceiving; look for loops; abstinence starts when I run out of porn memories*”). However, the “bottom-up” approach, which allows for the creation of codes and themes derived from the content [e.g., (36)], was also adopted for the data analysis. This resulted in the identification of new topics and the generation of new codes. These novel codes were read once again to develop potential themes (e.g., sexual objectification of women - “*when I use public transport and meet different women on the tram and start fantasizing about them in a sexual way*”). The themes were further sorted and refined in order to make them distinct and coherent (i.e., this step led to the specification of ways that the criterion of behavioral addiction may manifest, like the increasing amount of time or the increasing intensity of the sexual material), while the codes were allowed to be included in several themes at the same time. Finally, the bottom-up themes were re-analyzed in relation to the criteria of addictive behavior (e.g., sexual objectification emerged as part of withdrawal symptoms). The whole author team was involved in this step.

## Results

### Form of the Problem

In all of the participants, the out-of-control sexual behavior was related to the internet and there was no indication of offline out-of-control sexual behavior (however, five participants reported occasional visits to sex workers or serial infidelity - P3, P8, P10, P17, P18). The primary source of their problem was masturbation and online sex content consumption—primarily online pornography, although the majority of participants indicated the occasional excessive visiting of dating websites and cybersex via chats and video chats. Specifically, cybersex played a role in intensifying the experience when pornography alone was not viewed as exciting and rewarding enough, typically toward the end of a *session* (i.e., when ejaculation was demanded). There were various contexts for porn consumption (e.g., porn on a smartphone together with masturbation and quick ejaculation in a bathroom, repeated several times a day). However, the most prominent type of problematic behavior, in both the pleasurable as well as the destructive form, was a *session* where the person was alone, watching porn, and masturbating, yet trying to delay ejaculation for several hours. One of the perceived indications of addiction was an inability to resist the temptation to stay in the pleasurable state for as long as possible (i.e., not performing a simple quick masturbation). Concerning the form of the problematic behavior, we consider our sample to be homogenous.

### Development of the Problem

Porn consumption was originally a response to sexual urges. Over time, it became a dominant and more convenient activity than any other sexual practices. According to half of the participants, a precursor to their problematic behavior was excessive masturbation during adolescence (P3, P4, P9, P10, P12, P13, P14, P16, P17, P19, P21, P22). However, the problem emerged/started and was fully recognized much later, generally in the third decade of their lives (Mage of problem recognition = 26.05, SD = 5.39, Median = 25). Most of the participants reflected on the start and escalation of the problem during the first years after high school, when they had more time to be by themselves. Particularly those who transitioned to university claimed that the combination of (1) too much spare time, (2) an unorganized time schedule, (3) the need to be constantly on the computer, (4) periods of increased stress, and (5) having shallow social connections, all enhanced their consumption of online pornography and related sexual content. Over time, the participants did not know which other activity they could use to either fill their spare time or to cope with various feelings, like boredom, stress, and loneliness.

“Especially before exams, I felt anxious, in tension, stressed, you know? And then usually I was not able to concentrate, my mind was flooded with erotica. Then I was watching porn [and masturbating] really a lot, so I was exhausted both physically and mentally. And it is a vicious circle because that only starts more stress, shame, compunctions… I am not sure if I ever had any hobbies. So, it was the stress and boredom in college times, those were the roots” (P3).

Another pattern, while not exclusive to the previous one, was connected to the lack of an intimate partner and being overall unsuccessful on the marriage market (P10, P11, P21, P22).

“I was always very shy, it takes me a lot of energy to overcome my inner barriers to contact and talk to women. Till 30 I was simply not courageous enough to start any attempt and had no sex, till 30 I was just watching porn” (P11).

Four participants developed the problem along with problematic substance use–two with alcohol consumption (P13, P8) and two with methamphetamine use (P15, P23). Their sexual behavior was intensified by their attempts to stop the substance use and remained significant even after several years of substance sobriety.

“The progression was so that I had an issue with both, porn and alcohol, and then my wife didn't want to be with me. I wanted sex and she didn't. But I was unable to have proper sex anyway [issue with premature ejaculation and erectile difficulties]. I had a small studio where I masturbated every day after work for several hours. And I was scared that my wife will find out. And I ended up hospitalized, I was drinking really a lot. …After [divorce and successful treatment of alcohol addiction] I had only the porn” (P15).

With respect to the development of out-of-control sexual behavior, the course of the problem was gradual and represented a long-term issue that started, for various reasons, in young adulthood and plateaued as a lifestyle for years.

### Treatment Experience

There was no clear pattern for help-seeking behavior. Participants contacted professionals based on their availability, without differentiating their educational background (e.g., psychotherapy, clinical psychology, psychiatry, sexology). Therefore, the participants differed in their type of therapy. Some were prescribed serotonin-based antidepressants (P2, P12, P14), most underwent psychotherapy, which, in three cases, were years-long processes (P17, P19, P23). Two men did not recognize the problem themselves (P4, P6); their partners were dissatisfied with their intimate lives because those men preferred pornography to partnered sex and faced erectile difficulties. In many cases, the problematic sexual behavior was not the focus of treatment because the participants mostly asked for treatment for depression (P2, P12, P14), erectile dysfunctions (P9, P12), and the treatment of problematic substance use (P5, P8, P13, P15, P18), and not for out-of-control sexual behavior, *per se*. None of the treatments were, in the words of participants, labeled as successful. When the participants explicitly mentioned their problematic pornography use, their healthcare providers were seen to neither really understand the nature of the issue nor provide an atmosphere or discourse that would encourage the participant to extrapolate about the problem:

“I felt humiliated to express myself, but the psychologist seemed to feel even more shame than I did. I think she did not expect what would come. And the therapy totally failed in the effect” (P7).

As an opposite example, Participant 9 was staggered that the sexologist did not see anything wrong with excessive masturbation as “it is not harming anyone else so it is OK to continue.”

The majority of the participants had issues with low self-esteem, loneliness, and perceived unattractiveness. The psychotherapy usually focused on addressing those issues and did not take into consideration the sexual behavior. According to a number of participants (P5, P8, P13, P15, P18) their psychotherapy was successful when dealing with other issues (e.g., alcohol, methamphetamine use, gambling); however, because the pornography use was not addressed, that problem later intensified as a substitute. For example, Participant 10, who tried to overcome porn addiction by throwing out his computer started visiting pubs, drank alcohol, experimented with amphetamines, and gambled to fill the post-pornography void. This new behavior, however, was labeled by his treatment provider as the “real harm” and suggested buying a new computer to stay at home and be entertained, which created a relapse to porn addiction and also led to excessive gaming.

These cases show that some professionals were not prepared to work with this issue because they underestimated the addiction factors, like a relapse. However, participants themselves confirmed that they did not feel comfortable opening up about issues related to their sexual lives and sexual excesses. This was, for various reasons, including the sensitivity of the topic (i.e., feelings of shame), the desire to keep porn in their lives despite the many problems it was causing, and because other issues, like alcohol use, despite being minor, were seen as more harmful at that moment.

### Manifestation

The symptoms were studied in two ways: by applying a clinical AICA-C interview and by letting the participants talk about what they found significant and how the symptoms actually manifested. In AICA-C (using a scale of 1-5 with 5 representing the most intense occurrence of the symptom while 1 representing the symptom not being present at all), the most significant symptom was the loss of control (average score 3.95), followed by preoccupation (3.52), craving (3.39), negative consequences (2.91), tolerance (2.69), and withdrawal symptoms (2.08). These scores also roughly corresponded to the comments made during the respondents' narrations; however, mood modification (not included in the structure of AICA-C) was very significant and common as well.

#### Salience

This criterion (i.e., referring to the interference of the activity by fulfilling important goals and obligations, overpowering one's thinking and feeling in the form of cognitive preoccupation and craving) manifested in several ways. First, participants intensively spoke about their experience with a **cognitive preoccupation** with sexual thoughts and fantasies and a craving. In their narrations, craving and cognitive preoccupation were not separable. Some thoroughly described triggering situations that provoked craving and fantasizing–mostly upon seeing women on the street, at work, in shopping malls, or just in advertisements and on billboards (P1, P3, P6, P7, P8, P9, P10, P12, P14, P15, P16, P17, P18, P19, P20, P23):

“…during spring and summer, walking on the street was like browsing a catalog for pornography” (P23).

Other men, however, faced intrusive sexual thoughts despite the lack of sexually explicit cues and without any specific pattern (P1, P5, P7, P8, P13, P14, P20, P22, P23). Nonetheless, some had their mind suddenly “flooded” with sexual fantasies in non-triggering situations that were labeled as “times of emptiness”: situations where they were alone, bored, and doing repetitive work (P2, P3, P6, P7, P9, P10, P11, P14, P16, P20, P21) or when stressed, sad, in a bad mood, or generally down (P3, P6, P9, P10, P11, P16, P17, P18, P20). Salience was also intertwined with **ritualization**. Watching online pornography was anticipated as a habit before sleep, after work, and during free time (P9, P17, P19). Such situations developed into triggering cravings, as seen in an example of one participant who regularly experienced sexual fantasies and cravings during the last hour of the workday:

“I was just waiting for when it would be the time when I'd be able to turn on the internet. I was just looking forward to it and I was not capable of doing anything, no meaningful thing” (P9).

For most of the participants, the activity dominated their **lifestyle** and represented the only way they were able to spend free time (P1, P2, P7, P8, P10, P12, P13, P14, P15, P16, P17, P18, P19, P21, P23).

All other activities were sacrificed (P12, P13, P17, P21). Only one participant mentioned that he was freely able to switch to other leisure activities (P22) while several mentioned that they were not (always) bothered because it was a hobby (so that they looked forward to it) (P1, P10, P11), and two others were reconciled with the behavior (P4, P9).

“I was thinking why I like it so much when it is such a time-eater. But I realized it is a hobby like anything else. You also spend that time if you like fishing. It is a way of life” (P1).

#### Mood Management

Probably the most important reason or motivation for why the participants started and continued in their patterns of porn use was **pleasure-seeking**. Basically, all of the participants realized that engaging in online sexual activities provided them with a large amount of (albeit usually short-term) positive feelings, such as a “good feeling,” “pleasure,” “joy,” a “perfect escape from reality,” and the “pleasant feeling of being in a whirlpool”:

“It's like when, for example, I lay in a hot tub and I feel comfortable there and I stay there longer than I originally wanted to” (P1).

Another function of internet use for sexual purposes was to **counter the state of boredom**. This was an often-mentioned reason for porn use that preceded the start of problematic behavior. However, over time, the participants did not know how to spend their leisure time. Some participants explicitly described that there might be a bi-directional association between excessive pornography use and unstructured time, in that both may be the outcome and the origin of one another. In other words, poor time management in addition to procrastination could play a role in the development of out-of-control behavior.

“I had time again, a lot of free time, and there would be only one way to fill it. Because even if I spent 2 h with porn, I then had another 10 h where I often just had nothing to do... So what used to be, basically, a leisure-time or procrastinating activity at the beginning, became a stress-conditioned neurotic obsession” (P4).

Over time, the positive motivation for the behavior started to be overshadowed by its use as a coping strategy to **avoid negative emotions**.

“Then I was just horribly frustrated with my life, every evening I felt like that. So I was just looking forward to the escape, to experience at least something nice” (P15).

Many participants acknowledged that they used online pornography as an escape from stress (P3, P4, P12, P13, P14, P16, P20); as a way to deal with conflicts with partners and colleagues at work (P2, P6, P9, P11, P12, P15, P17, P18, P20, P21); as a way to soothe bad moods and general life dissatisfaction (P3, P6, P5, P8-19, P21); as a way to cope with loneliness (P2, P4, P7, P20). However, a few found this to be an inefficient and counterproductive strategy because they felt even worse afterwards (P1, P3, P5, P13, P22):

“… and after three-four hours [of watching porn] the feelings of despair kick in that I totally wasted myself and everything... I simply like it too much, I want the pleasure, I want the porn, but then it is also a life failure” (P22).

#### Loss of Control

Loss of control represented the most important characteristic of the problematic behavior. Except for Participants 2 and 6, who generally believed that they had their excessive pornography use under control, all of the other participants expressed they will always “lose the battle.” There were two main patterns for the loss of control. First, there was a **loss of sense of time and self** during sessions, a tendency to stay much longer with the pornography than originally intended, and total immersion (P1, P3, P5, P7, P10, P11, P12, P13, P19, P20, P22, P23):

“I just opened the computer, just to read emails, and then I stayed the whole night watching and masturbating and, at the end, I had no idea how that actually happened” (P10).

Some participants even called this tendency “insanity,” a “state of madness,” and “total obsession” and they felt the uncontrollable need to do it as much as possible (P4, P7, P8). Often, they struggled with whether to open the computer at all, but they knew that when they had such an idea, there was no way to resist it. The tendency to struggle with the intention only increased the appetite and desire (P19, P20, P21). And even with the intention “just to have a look” they ended up watching porn as much as possible, unless external factors, such as family, school, work obligations, or strict time schedule would not allow it. External factors (i.e., family, school, and work obligations, strict time schedule) were generally very important; otherwise, the participants felt unable to manage themselves:

“I really did not like weekends. From Monday to Friday, I was in school, I had some obligations, and there was less room for pornography or masturbation or some sort of fantasies. And then I just feared the weekends” (P14).

The second type of loss of control was a **relapse** into the behavior after a period of relative control. It showed the inability to abandon the habit for good. All participants experienced at least some relapses and the majority of them experienced many intensive relapses (P1, P3, P4, P6, P8, P9, P10-17, P20-23). Especially after a period of abstinence, some participants had extended sexual sessions and quickly returned to the consumption of hard pornography in order to “catch up on” everything they had missed:

“And when there is that day [of relapse] that you are alone at home, when you start the day like this, you can bet good money that the day will go to hell and that you won't do what you wanted because you will have to repeat it maybe three, four times [sessions that are each several hours long]” (P32).

In the majority of cases, the (temporary) abandonment of the behavior was natural (i.e., the pornography started fading in its effects), until it faded enough that the participants started to feel the need to renew the sexual images in their mind (i.e., see the part on withdrawal symptoms). Interestingly, some participants had experience with the impulsive termination of the behavior (e.g., Participant 9 once destroyed his computer and later cut through the internet cables).

#### Conflicts and Negative Consequences

Participants were clear about the problems that their out-of-control behavior caused. At the intrapsychic level, more than half of the participants spoke about self-disdain and self-degradation to the point that they stopped respecting themselves. Typically, they had feelings of self-disgust, shame, and even suicidal thoughts (P1, P2, P4, P5, P10, P11, P12, P14, P16, P18, P19, P20, P21, P23):

“I cried so many times because of it, and then I did not know at all what I should do” (11). Moral conflicts due to religious beliefs were reported in some participants as well; however, they were not felt to be as major as other conflicts (P5, P7, P9, P14) and they were mentioned only when directly asked about them.

For some, this behavior led to a stagnation in their career (P1, P2, P7, P12, P13, P17) and general life stagnation, neglected family life, missed life opportunities, and feelings that their life was being wasted (P2, P3, P8, P17, P18, P19, P20). Excessive behavior (especially in the form of sessions) led to significant weariness, exhaustion, and lack of sleep (P3, P8, P9, P11, P14, P15, P18, P22, P23).

“It was several times a day [sessions that took 2 h each] four-five times a day was the peak, and I was simply exhausted, the penis was used so much that it pained a lot, but I continued because I wanted it [to stay with the porn], you simply must continue, you must, but the body says no” (P14).

From the sexual point of view, the majority of the participants confirmed combinations of various problems, including penis pain due to prolonged masturbation sessions, erectile dysfunctions, and premature ejaculations due to the desensitization of their sexual stimuli, and the general loss of interest in normal sex (P1, P2, P4, P9, P10, P12, P13, P14, P16, P17, P20, P23). Some of those who were in a long-term partnership reported conflicts with their spouses, especially due to their higher preference for virtual sex (P2, P6, P7, P8, P9, P11, P14, P18, P23) or because they developed a preference for coercive sexual practices (P13, P15, P18).

“I had an erectile issue at that time. The andrologist examined me and there was nothing physiological. I had a partner, and she thought she is not attractive or that is her fault. And the relationship stopped working. But it was just the porn, I was used to porn and the real sex simply was not what could arouse me” (P9).

Those in relationships reported that the porn use isolated them from their partners and that they were no longer able to experience intimacy and closeness in their relationships. The main and very strong pattern of negative effects was that an overwhelming majority of the participants struggled with reducing women to sexual objects:

“Today I notice other things about women than I did before. Because [the addiction] always wanted to see just that dirty stuff, to tell you the truth. But today, as though I am getting better, I already notice other things about a woman, things like the eyes, the smile…” (P3).

Being able to acknowledge women as more than sexual objects was considered to be a sign of recovery by the participants.

It is important to note that negative consequences were experienced as long-term issues that were possible to keep secret for a very long time (i.e., in the words of participants as “hidden,” “invisible addiction”). The destructive potential of the behavior was felt more in retrospect (as a “wasted life”) rather than as an acute state that would significantly facilitate a search for help.

#### Tolerance

There were three participants who reported no form of tolerance (P2, P6, P11). However, the majority of participants had experienced some form of increasing tolerance in their behavior. These took various forms. It manifested as the **growing amount of time** spent on online sexual activities (P5, P7, P8, P9, P10, P12, P13, P14, P15, P17, P18, P19, P21, P23). The respondents prolonged the sessions (typically from 1 h to more than 8 h) and/or they included more sessions into their daily routine, like very early in the morning, which was usually preferred:

“And it just escalated, so that I was looking more often for certain movies. In the end, I set my alarm clock so that it would wake me at three in the morning, so that it woke me up because I knew that I just had to” (P7). Increasing time usually escalated to the point that the participants become satiated, so they left the behavior for some time only to return to it after some time of control (usually a matter of weeks).

Building up a tolerance to online pornography also manifested as the **growing intensity of the sexual material**. This could be partially explained as desensitization:

“It is a thing that always needs more and more, because those pictures stop being really, like, hot. They stop working, and a person needs a stronger stimulus” (P20). There were several types of progression-erotic pictures had to be substituted with more sexually explicit materials, mainly video chats (i.e., cybersex) and the communication in erotic chats also became more and more obscene. Also, the content, classic heterosexual vaginal intercourse, was no longer attractive. With growing frequency, the participants searched for hardcore porn sites with more intensive stimuli (P1, P10, P12, P13, P14, P15, P16, P18, P20, P22). This even manifested as a greater openness to exposure to paraphilic-focused sexual and fetishist materials, typically content included zoophilic, hebephilic, rape, coercive, and generally sadomasochistic materials (P3, P10, P12, P13, P14, P15, P18, P20). However, when inquiring about the content, participants were generally unwilling to share this information and considered it to be a sensitive issue. Often, this unceasing need for extreme stimuli resulted in strong negative feelings:

“So then I was really disgusted by what I was watching, because it was still harder and it just did not often bring that effect” (P13). It must be noted that this progression (to fetish or extreme and paraphilic materials) stayed within the session and did not transform into long-term changes in sexual preference. Subjects described that, during porn-masturbation sessions, their psyche was in a state of madness, in which they were incessantly searching for new material on the internet, clicking on more and more videos. Also, to reach ejaculation after an hours-long session of masturbation, they needed stronger than usual stimuli.

“Yeah, it was that it simply was not enough and, definitely, that I was not excited, so I was looking for more of what excites me. And still, the extra was too little, so I still searched for what would excite me” (P12).

In some cases, **pushing the boundaries** in physical contact also characterized increasing tolerance. Some participants (P1, P9, P15, P17) pushed the boundaries for the sexual activities they would practice, and they were prepared to take greater risks (e.g., compromise their anonymity in cybersex). They even became afraid of where this adventurism would end up:

“You allow more, you are more daring, you let yourself do more than you had done before. I watched pornography in front of my wife. I masturbated in front of her, but, of course, without her seeing it; this is not something I would have done in the beginning” (P7). “I was doing cybersex sometimes, but then I also started visiting videochats that were not erotic, searching for girls and masturbated on camera” (P1).

#### Withdrawal Symptoms

The study identified various **acute unpleasant symptoms** when the participants had to terminate the activity and especially when they could not, or did not want to, perform the activity for some time. However, it must be said that most participants found these symptoms rather mild and controllable. One of the reasons for the rare experience of withdrawal symptoms was that masturbation was found to be easily performable when needed and thus negative states were easily avoidable (P1, P7, P12, P17, P20, P21). They could masturbate with the use of their memories of the consumed porn or their imagination about sexual objects (mostly women met on a street). In general, symptoms included increased emotionality, like nervousness and the inability to focus (P2, P3, P5, P7, P8, P12, P13, P14, P15, P16, P18, P19), and increased irritability/frustration (P4, P7, P8, P10, P12, P13, P14, P15, P16, P18, P22, P23), which emerged when they could not watch porn, could not find an adequate sexual object, and had no privacy for masturbation.

“I tried to not do it [neither watching pornography nor masturbating]. Well, of course, this resulted in problems in my relationship. I was feeling unbelievable, like a surge of anger. And I was smashing things and I blamed my wife for everything possible…” (P15).

Rare symptoms included intensive apathy (P10), seeping difficulties (P9), permanent sexual arousal (P11), and various bodily feelings (e.g., chills, sweating, headache, sickness), likely as a result of somatization (P19). However, some respondents expressed doubts that acute withdrawal states do really exist (P15, P16, P17); according to them, negative states were experienced because they did not, or could not, use porn and masturbation as coping mechanisms.

Apart from acute withdrawal states, respondents also described experiencing mental/cognitive states that resulted from long-term abstinence from pornography and that could be understood as **pre-relapse states**. First, there was a phenomenon of **fading memory images**, where they were no longer able to recall the exact images that used to arouse them, and when they yearned to look at any sexual object offline in order to refresh their memory (P3, P4, P9, P10, P12): “But the fight [for abstinence] lasted half a year. Gradually, I suddenly forgot how it actually looked, I mean all that pornography. Hell, what she (looks) like, what was in that movie and everything?... I have almost no recollection right now, what will make me happy, will I ever be happy?” (P3).

Many participants described **intensive longing** — a strong desire to recall sexual images and reach exposure to sexually explicit content (P3, P4, P5, P7, P9, P10, P13-17, P19, P20). The lack of sexual memory generated a specific compensatory behavior. Nearly half of the participants talked about using **exploitative staring** (P3, P7, P12, P13, P15, P16, P17, P18, and P20). This can be understood as a substitution strategy that is based on searching for any kind of sexual object (i.e., women in a public space). This type of objectification of women is similar to what we described in the salience section above. However, in this case, it is deliberate behavior (e.g., visiting swimming pools, bars, other places where they could expect to see women):

“I remember when I was without pornography. It was not only the most attractive woman that I was staring at. I tried to make the most of everything, to get enjoyment from it. I seriously searched so much for anything that I was staying on the balcony to search if I would see any woman down below” (P16). This excerpt suggests that, during a period of abstinence, the participant's mind missed being flooded by pornographic images. Therefore, this participant tried to get as much as possible from each random potentially sexual object in order to feed his fantasy and mind.

## Discussion

The aim of this qualitative study was (1) to provide insight into the experience of 23 men who sought help for their problematic sexual internet use and (2) to enhance our understanding of whether the phenomenon should fall within the hypersexual, compulsive-impulsive, or addictive spectrums of disorders. In this respect, the patterns of the problematic behavior, the manifestation of symptoms, and the development of the issue over time were analyzed and supported, especially the relevance of the addiction conceptualization.

The problematic behavior typically included excessive masturbation while watching porn for several hours and repeated several times a week or day, and occasionally amended with other online sexual activities. All of the participants (except for four) fulfilled all of the criteria for addiction, including the signs of tolerance and withdrawal symptoms, which indicates that the addiction model is useful for the understanding of the phenomenon. This finding corroborates other recent studies that have reached similar conclusions [i.e., the addiction models seemed to fit the description of the symptoms associated with PSIU; (4, 41)]. Nonetheless, it must be noted that our support of the addiction model does not automatically disregard other models, either hypersexuality or CSBD. In fact, the core criteria for all the three models—salience, the loss of control (including relapses), and consequent problems—were experienced very strongly by the participants and, in addition, these reached the highest average scores in the AICA-C clinical interview. In this respect, all three models seem to be relevant. However, the significance of mood management indicated support for the hypersexuality and addiction models more than the CSBD. The gain of positive feelings, ranging from excitement and pleasure to countering the states of boredom, was reported as the primary motivational factor in the engagement in the problematic sexual behavior, despite the adverse consequences. The activity was also used as a means for coping with negative mood states (e.g., stress, anxiety); however, the importance of this purpose developed over time as a result of the excessive engagement in the activity. This development—the gradual shift from primarily a gratifying use of pornography to a compensatory use—has been described in the I-PACE Model for behavioral addictions (42) and further supports the validity of the addiction model in our study.

The notion and the existence of the criteria of withdrawal symptoms and tolerance have been criticized and doubted in behavioral addictions, in general (27, 34), and particularly with respect to excessive sexual behavior (26). In our study, experience with these symptoms was common. The tolerance manifested as increasing time devoted to the problematic activity, increasing willingness to push the boundaries of what would be considered safe, and especially as the increasing roughness of the consumed erotic materials. The erotic content sometimes reached the levels of being close to paraphilic content. However, the participants themselves did not consider themselves to be paraphilics nor that the paraphilic content (i.e., eliciting sexual arousal patterns that focus on non-consenting others) was their sexual preference. Furthermore, the periods of increased engagement in the activity were regularly superseded by the periods of the diminished effectiveness of the erotic materials used to induce arousal. This effect is labeled as a temporary satiation (39). Concerning the withdrawal symptoms, they manifested as mild distress–nervousness, irritability, and, occasionally, physical symptoms due to somatization. However, as compared to other symptoms, withdrawal symptoms were not seen as significant or disturbing. Moreover, it was not clear to what extent the symptoms were built up because porn could not be used as a coping mechanism for negative states of mind. In this respect, the criticism of withdrawal symptoms in behavioral addictions is partially justifiable (26). However, we identified another form of potential withdrawal that we could not detect within the literature. During the temporary satiation phase, when the erotic images faded from memory, the participants started to feel the distress and the urge to renew them. In most of the participants, this usually resulted in increased sexual-objectification behavior (i.e., searching for scantily dressed women, staring at them and at their sexual parts when possible). These acts generally signified a phase that put male sex addicts at risk of relapse.

According to some researchers, the symptoms approach in behavioral addictions is problematic. Instead, they define the addiction as (1) functional impairment and (2) persistence over time (34). Both of these conditions were fulfilled in our study–the problems caused by engagement in the activity were common (together with the loss of control and salience/craving). The participants ascribed their excessive online pornography use to many adverse impacts on their mental and physical health, as well as on their personal, family, and work life. Furthermore, their intimate and sexual lives were also negatively affected (e.g., by erectile difficulties, loss of interest in partnered sex, inability to share intimacy with their life partners). The issue itself was experienced for a long time−10 years on average–culminating in early adulthood and basically plateauing afterward. The fact that the issue is deeply embedded into the participants' lifestyles points to the targeting of these problems in potential intervention.

There are several practical reasons that signify the importance of understanding PSIU as a behavioral addiction. First, there was high comorbidity with other conditions, especially with other addictive behaviors, including alcohol and amphetamine use, gambling, and excessive computer gaming. Since the co-occurrence of addictive behaviors is common (40), the other (non-sexual) conditions could be seen as more harmful by the medical professionals, and the treatment targeted them instead of the sexual behavior (despite the fact that the sexual behavior was the main condition). Second, the consequences of the porn use were not felt by the participants as immediately threatening and harmful (unlike the use of methamphetamine or gambling) and they accumulated negative effects slowly over a long time period. Third, the shame surrounding this phenomenon may be a significant obstacle in treatment. The sensitivity of the issue discouraged the participants from fully disclosing their condition to the health professionals. Instead, they waited for the professional to address the issue, which often did not happen, raising the question of whether training professionals in sexual issues in general, and specifically potential sex and porn addiction, would improve their clinical practices. Although there is evidence that points to the role of moral and religious incongruence in the false indication of sex and porn addiction (30), our study showed that the feelings of shame may also have different origins. The negative feelings stem from the intensity of the behavior and the roughness of the consumed content (e.g., human-animal sex, rape). Since paraphilia is generally considered to be exclusion criteria (8, 11, 14), the presence of paraphilic or near paraphilic content may be confusing in diagnostics and should be further explored. Some studies reported the co-occurrence of paraphilic content consumption and porn addiction (19); however, that is usually explained by the compensation of unfulfilled sexual fantasies (43). In our study, it was connected to the effect of tolerance and desensitization.

Some limitations of the study should be noted. First, the findings are limited by what the participants shared with regards to their sexual lives and the content of the consumed online pornography. The participants were largely unwilling to talk about the content of the materials they were consumed and they were also uncomfortable about discussing the extent of their behavior. Second, the sample included participants who were members of Sex Addicts Anonymous and Sexaholic Anonymous, whose narration of their stories could have been more influenced by addiction models, which are at the core of the 12-step program (44). Third, our sample included only men. Although literature suggests that this phenomenon is more common in men (45), there are studies that identified the specifics of sex addiction in women (46). Similarly, our sample predominately included heterosexual men, while the non-heterosexual orientation has been identified as an important risk factor for problematic sexual behavior (47). In general, women and non-heterosexuals within PISU are under-researched and future studies should focus to fill this gap. Fourth, the AICA-C clinical interview has not previously been used and calibrated in the Czech language and its coding was done by only one researcher, thus the inter-rated reliability could not be assessed. Lastly, the sample included participants who predominately had a problem with pornography use. Other forms of online sexual behavior like cybersex and visiting dating sites were minor in our study and problematic offline sexual behavior was not found. Thus, our study is applicable only for (1) online pornography use and not to other forms of sexual behavior, and (2) the use is intensive enough that the participants decided to seek professional help.

We acknowledge that the criticism of the usage of addiction terminology in relation to common or just vaguely problematic porn use may be justifiable (e.g., 28); however, this study shows that, in the case of help-seeking men and their problematic pornography use, the addiction model of the available conceptualizations was the most useful to describe the condition in the present sample.

## Data Availability Statement

The raw data supporting the conclusions of this article will be made available by the corresponding author, without undue reservation.

## Ethics Statement

Ethical review and approval was not required for the study on human participants in accordance with the local legislation and institutional requirements. The patients/participants provided their written informed consent to participate in this study. Written informed consent was obtained from the individual(s) for the publication of any potentially identifiable images or data included in this article.

## Author Contributions

AŠ conducted the interviews and did the data analysis supervision. LB conducted the analysis and wrote the first draft. LB, AŠ, MD, KŠ, and KW interpreted the results and edited the draft. All authors contributed to the article and approved the submitted version.

## Conflict of Interest

The authors declare that the research was conducted in the absence of any commercial or financial relationships that could be construed as a potential conflict of interest.

## Publisher's Note

All claims expressed in this article are solely those of the authors and do not necessarily represent those of their affiliated organizations, or those of the publisher, the editors and the reviewers. Any product that may be evaluated in this article, or claim that may be made by its manufacturer, is not guaranteed or endorsed by the publisher.
